# A case of NMDAR Encephalitis with muscular pain as the main presentation

**DOI:** 10.1186/s12883-024-03652-w

**Published:** 2024-04-27

**Authors:** Baizhu Li, Xiuli Shang

**Affiliations:** The First Affiliated Hospital of China Medical University: The First Hospital of China Medical University CHINA, Shenyang, China

**Keywords:** Persistent somatoform pain disorder, Autoimmune encephalitis(NMDAR), Case report, Anti-NMDAR encephalitis, Early diagnosis

## Abstract

**Background:**

Persistent somatoform pain disorder (PSPD) is often the initial diagnosis in patients seeking treatment in psychiatric departments, making it challenging to consider organic nervous system diseases. However, autoimmune encephalitis can present with atypical initial symptoms, leading to misdiagnosis or missed diagnosis. Lumbar puncture, with antibody support, plays a crucial role in diagnosing autoimmune encephalitis.

**Case presentation:**

This report describes a 40-year-old male adult patient who was initially diagnosed with persistent somatoform pain disorder in 2022. The patient reported a reduction in pain while resting on his back. There were no fever or relevant medical history. Despite 8 months of symptomatic treatment, the symptoms did not improve. Moreover, the patient developed confusion, gibberish speech, non-cooperation during questioning, and increased frequency and amplitude of upper limb convulsions. Lumbar puncture revealed elevated protein levels and protein-cell dissociation. The autoimmune encephalitis antibody NMDAR (+) was detected, leading to a diagnosis of autoimmune encephalitis (NMDAR).

**Conclusion:**

Autoimmune encephalitis (NMDAR), starting with persistent somatoform pain (PSPD), often presents with atypical symptoms and can be easily misdiagnosed. Therefore, it is important to consider the possibility of organic nervous system disease in time, and to test serum or cerebrospinal fluid antibodies to rule out organic nervous system disease after symptomatic treatment of mental disorders is ineffective. This approach facilitates the early diagnosis of autoimmune encephalitis and other underlying organic neurological disorders.

## Introduction

Autoimmune encephalitis, especially NMDAR (N-methyl-D-aspartate receptor) encephalitis, is an autoimmune disease that affects the brain [1]. It is characterized by the presence of autoantibodies targeting NMDAR receptors in the brain, leading to central nervous system inflammation and dysfunction [2] .Psychiatric symptoms have been reported to accompany autoimmune encephalitis and are often the initial sign of the disease [3].

Recently, we found that persistent somatoform pain disorder is also very likely an early manifestation of autoimmune encephalitis. This paper reports a case of persistent somatoform pain disorder diagnosed as autoimmune encephalitis (NMDAR).

## Case

On March 2, 2022, a 40-year-old male adult patient presented with unexplained muscle pain in the extremities, abdomen, and lower rib margins. The pain was predominantly experienced during vigorous physical activity, with the lower limbs being more severely affected. The patient reported a tingling sensation in the feet while walking, which worsened with increased activity. He reported a reduction in pain while resting.The patient was treated with pregabalin 150 mg daily.

In April 2022, in addition to the muscle pain, the patient developed symptoms of fatigue, weakness, and decreased appetite. The patient was changed to duloxetine 60 mg and had a limb twitch of the left upper limb during sleep, but only once and informed by the family.

On September 19, 2022, the patient was admitted to the psychiatric department of our hospital with severe muscle pain in all four limbs as the main complaint. The patient denied any history of cold or other related illnesses. In the absence of any identifiable trigger, muscle pain persists, worsens with activity, and causes tingling when walking.The patient also experienced fatigue and a sense of exhaustion. However, when lying flat, the patient did not experience somatic pain, leading to a diagnosis of persistent somatic-type pain disorder. Treatment with duloxetine 120 mg was started but did not provide adequate relief. During the course of treatment, the patient’s symptoms worsened. The family reported absence seizure of five seconds at a time with no memory. There was no tongue biting, urinary and fecal incontinence. However, laboratory investigations including CK-MB, rheumatological, coagulation, complete blood count, liver and kidney function tests, muscle calcium protein, electrolyte levels, erythrocyte sedimentation rate, thyroid function, calcitonin, tumor markers, antiphospholipid antibodies, electrocardiography, brain computed tomography (CT), magnetic resonance imaging (MRI) Table 1 scans of the brain, cervical, thoracic, and lumbar spine, and electroencephalography, PET-CT examination Electromyography (EMG) and nerve conduction velocity (NCV) testing did not reveal any significant abnormalities.

**Table 1:**
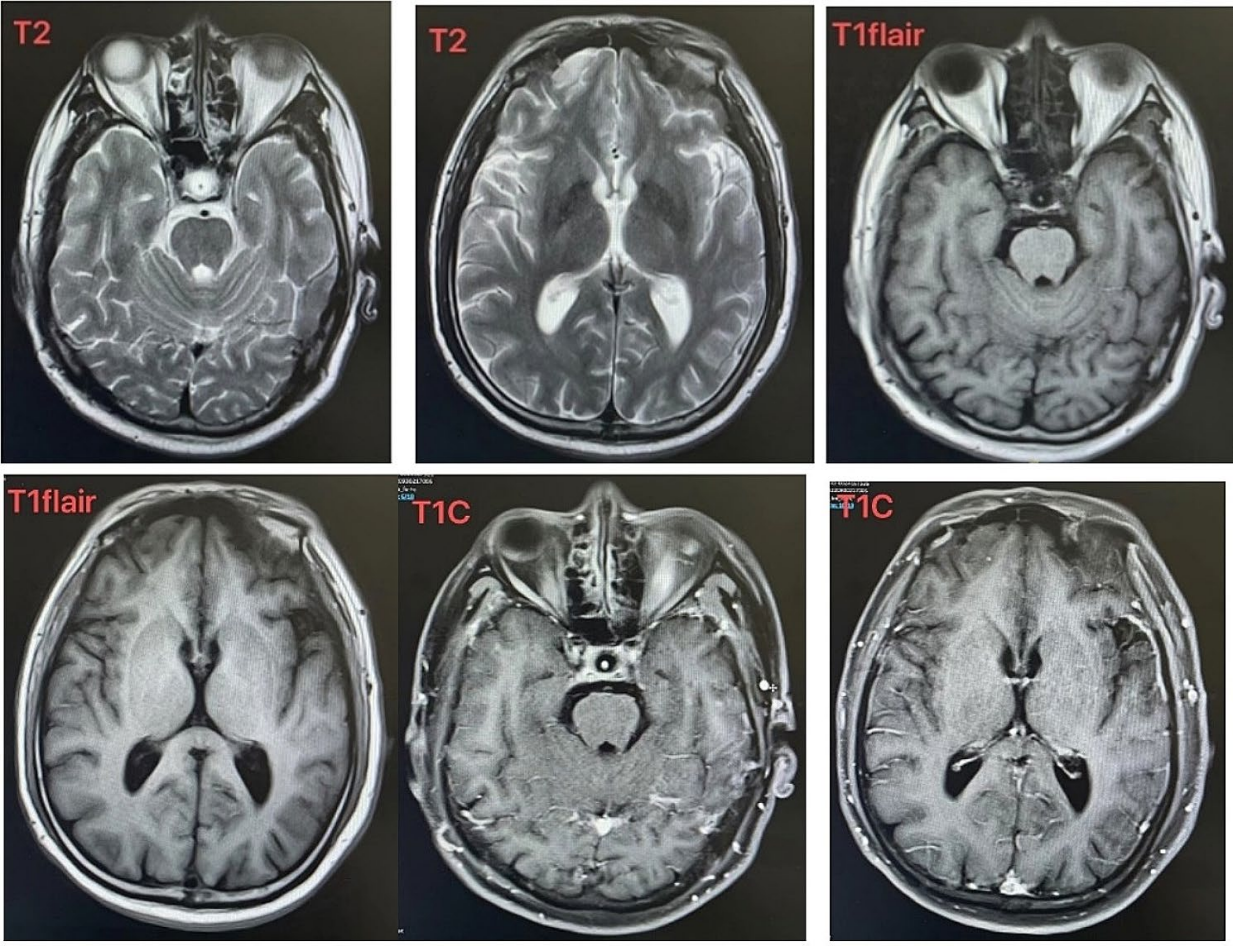
Table 1 Cranial MR Plain scan (3.0T) showed ethmoid sinus cyst. The rest showed no obvious abnormalities

The patient’s symptoms did not improve significantly during the use of duloxetine 120 mg; instead, the symptoms worsened. The pain intensified while standing and diminished while lying down, with widespread pain throughout the body, and pronounced tenderness upon touch. Additionally, the patient developed confusion, incoherent speech, poor response to questions but did not exhibit fever or other related symptoms. The frequency of tic attacks in the left upper limb only increased to once a half month, which occurred during sleep. The patient’s absence seizure occurred once a month and lasted 10 s. The patient was treated with sodium valproate 500 mg twice daily, but the symptoms did not improve.

Upon initial admission to the psychiatric department, the patient’s somatic pain score [4]was 3, which increased to 6 with pregabalin 150 mg treatment and further increased to 8 after switching to duloxetine 120 mg. Subsequently, on November 11, 2022, the patient underwent further evaluation in the neurology department. At the time of admission, the somatic pain score [4] was 8 and 21 on the Montreal Cognitive Assessment (MOCA) [5]. After the neurology department confirmed the diagnosis and provided appropriate treatment, the patient’s somatic pain score [4] decreased to 0, while the MOCA [5] score improved to 25. Clinically, the patient exhibited confusion, incoherent speech, poor response to questions, dizziness, and nausea before treatment. Following treatment, the patient regained normal communication skills and clarity of thought.

Laboratory investigations revealed the following results: the initial cerebrospinal fluid (CSF) analysis showed elevated protein levels (1445 mg/L↑)(reference range:120-600 mg/L), decreased chloride levels (112mmol/L↓) (reference range:120-132mmol/L), low glucose levels (2.9mmol/L) (reference range:2.2-3.9mmol/L), and a cell count of 0 × 10^6/L(reference range:0–8 × 10^6/L). The patient tested positive for NMDAR antibodies in both the CSF and serum (NMDAR+:1:32). Other tests for demyelinating antibodies, paraneoplastic antibodies, and peripheral neuropathy antibodies were within normal ranges. In the follow-up CSF analysis, the protein levels decreased (1322 mg/L↑) (reference range:120-600 mg/L), chloride levels further decreased (117mmol/L↓) (reference range:120-132mmol/L), glucose levels increased (3.0mmol/L) (reference range:2.2-3.9mmol/L), and the cell count increased to 3 × 10^6/L(reference range:0–8 × 10^6/L). The patient tested positive for NMDAR antibodies (NMDAR+:1:10) in both the CSF and serum. Other tests for demyelinating antibodies, paraneoplastic antibodies, and peripheral neuropathy antibodies were within normal ranges. Additional laboratory investigations did not reveal any significant abnormalities.

Treatment: The initial treatment consisted of oral folic acid tablets (5 mg, three times a day), oral methylcobalamin (0.5 mg, three times a day), and oral vitamin B1 (thiamine) tablets (one tablet, three times a day). Additionally, vitamin B1 and methylcobalamin were administered via intramuscular injections once daily. After observing the patient for five days, methylprednisolone was given as pulse therapy at a dose of 0.5 g for seven days. Patients were treated with 250 mg hormone pulse for 3 days.The patient was started on 80 mg oral methylprednisolone tablets.Potassium and calcium supplements were provided to address electrolyte imbalances, and measures were taken to protect the stomach.

As a result, the patient experienced a reduction in self-felt pain after 7 days of hormone administration. After half a month of treatment, the patient’s condition improved significantly. Before discharge, the patient reported no pain, returned to normal function, and resumed daily activities without any significant limitations, and the patient’s pain scores, cerebrospinal fluid titers, and serum titers were reduced on review. The patient had no recurrent pain and related symptoms during telephone follow-up 6 months after discharge.

## Discussion

The N-methyl-D-aspartate receptor (NMDAR) plays a crucial role in the normal physiological and pathological states of the brain [6]. Anti-NMDAR encephalitis is a characteristic neuroimmune syndrome where the patient’s own antibodies bind to the extracellular epitopes of NMDAR receptors, leading to impaired NMDAR function. This condition can affect males and females across all age groups and is characterized by symptoms such as psychiatric disturbances [7].

In the early inflammatory phase of anti-NMDAR encephalitis, patients may have a variety of symptoms, including epilepsy and psychiatric symptoms. At present, most studies focus on psychiatric symptoms as a rare early manifestation of autoimmune encephalitis (NMDAR). However, there are no reports in the literature related to NMDAR and persistent somatoform pain disorder [8].

In this case we considered persistent somatoform pain disorder as an early rare manifestation of anti-NMDAR encephalitis. However, this rare manifestation is easily misdiagnosed as pain symptoms caused by other causes. This patient had a history of tic attacks in the left upper limb and absence seizures lasting 5 s. Although the possibility of epilepsy was first considered, an electroencephalogram was normal, and antiepileptic therapy did not work. Thus, psychogenic nonepileptic seizures (PNES) were initially considered by psychiatrists. Therefore, when evaluating the patient, the physician should be aware of this abnormal pain pattern and perform a comprehensive evaluation when symptomatic treatment of pain is ineffective, while considering other symptoms and signs.

The association between anti-NMDAR encephalitis and persistent somatic symptom disorder (chronic pain) is relatively rare. However, this study mentions the occurrence of persistent somatic symptoms, including chronic pain, in patients with anti-NMDAR encephalitis. This may be related to the antibody-mediated attack on NMDAR receptors in the brain.

NMDAR receptors play a crucial role in signaling between neurons and are involved in pain transmission and regulation. When the patient’s immune system produces antibodies against NMDAR and attacks the NMDAR receptors in the brain, it can disrupt and amplify pain signals. This abnormal transmission may have significant implications in terms of neurotransmitter release and synaptic plasticity, leading to the manifestation of persistent somatic symptoms, including chronic pain [9]. Additionally, anti-NMDAR encephalitis can trigger immune-mediated inflammatory responses, leading to inflammation and damage to the neural tissue [10]. This inflammatory response may trigger abnormal excitability of peripheral nerve fibers, further exacerbating the symptoms of persistent somatic symptom disorder, including chronic pain.

Misdiagnosis or missed diagnosis of autoimmune encephalitis can have severe consequences, including disease progression and irreversible neurological damage due to delayed treatment. To address this, a multidisciplinary approach involving psychiatrists, neurologists, and other specialists is crucial for a comprehensive evaluation and accurate diagnosis [11]. As a rare early manifestation of autoimmune encephalitis (NMDAR), persistent somatic symptom disorder (chronic pain) should be highly suspected by clinicians. In the presence of other clues and without significant improvement of symptoms after symptomatic treatment, serum or cerebrospinal fluid antibody detection should be performed to exclude organic nervous system diseases, prevent misdiagnosis and missed diagnosis, and carry out targeted treatment as early as possible [12].

## Conclusion

Here, we report a case of autoimmune encephalitis with persistent somatoform pain disorder as the initial symptom. The time from onset to resolution was 8 months, and we followed the patient for up to half year. This patient’s initial symptoms were purely somatic - pain - and thus difficult to distinguish from common psychiatric disorders. It is hoped that through the warning of this case, clinicians should pay attention to patients with pain onset, so as to better identify and intervene in the early stage of organic nervous system diseases such as autoimmune encephalitis. Autoimmune encephalitis should be considered in the differential diagnosis in a patient presenting with pain as a somatic symptom, and this case is important in showing that neurologic symptoms can begin after the somatic symptoms of autoimmune encephalitis. Therefore, in the process of evaluating patients with such somatic symptoms, It is particularly important to use antibody detection and other methods to further rule out organic nervous system diseases after symptomatic treatment of somatic symptoms is ineffective and suspected neurological positive signs appear. Early detection, early diagnosis and early treatment of such diseases can avoid greater harm.

## Data Availability

Data is provided within the manuscript.
